# Forecasting Network Interface Flow Using a Broad Learning System Based on the Sparrow Search Algorithm

**DOI:** 10.3390/e24040478

**Published:** 2022-03-29

**Authors:** Xiaoyu Li, Shaobo Li, Peng Zhou, Guanglin Chen

**Affiliations:** 1 College of Computer Science and Technology, Guizhou University, Guiyang 550025, China; gs.xiaoyuli20@gzu.edu.cn; 2 State Key Laboratory of Public Big Data, Guizhou University, Guiyang 550025, China; 3 College of Mechanical Engineering, Guizhou University, Guiyang 550025, China; pzhou@gzu.edu.cn (P.Z.); gs.glchen21@gzu.edu.cn (G.C.)

**Keywords:** hyperparameter optimization, network traffic, prediction

## Abstract

In this paper, we propose a broad learning system based on the sparrow search algorithm. Firstly, in order to avoid the complicated manual parameter tuning process and obtain the best combination of hyperparameters, the sparrow search algorithm is used to optimize the shrinkage coefficient (r) and regularization coefficient (λ) in the broad learning system to improve the prediction accuracy of the model. Second, using the broad learning system to build a network interface flow forecasting model. The flow values in the time period [T−11,T] are used as the characteristic values of the traffic at the moment T+1. The hyperparameters outputted in the previous step are fed into the network to train the broad learning system network traffic prediction model. Finally, to verify the model performance, this paper trains the prediction model on two public network flow datasets and real traffic data of an enterprise cloud platform switch interface and compares the proposed model with the broad learning system, long short-term memory, and other methods. The experiments show that the prediction accuracy of this method is higher than other methods, and the moving average reaches 97%, 98%, and 99% on each dataset, respectively.

## 1. Introduction

The number of cloud platform users has increased in tandem with the development of internet technologies. In the context of high concurrency and limited cloud platform resources, how to reasonably allocate resources is one of the problems studied by cloud platform managers [1]. Forecasting the traffic of the cloud platform network interfaces is an effective way to achieve reasonable resource allocation: by predicting the traffic of each interface in the future, judging its resource demand, and accordingly allocating resources and planning the network to achieve a dynamic allocation of resources with the number of requests and achieve load balancing [2]. However, with the rapid increase of cloud platform access, scholars extracting internet traffic features for network traffic modeling and prediction not only have to consider its complex characteristics such as nonlinearity and multi-scale but also face the problems of decreasing prediction accuracy and increasing resource consumption caused by the increasing data scale. Therefore, the research of high-speed, high-efficiency, and high-precision network traffic prediction methods can not only further optimize and improve network resource provisioning, planning, and network security but also be extremely significant for the development of the internet and its good integration with other industries.

Network traffic forecasting belongs to the field of time series forecasting. The types of flow prediction methods include traditional statistical analysis [3,4] and machine learning. Traditional statistical analysis uses statistical and mathematical methods to make speculations and estimates on the development trend of internet traffic in the future period, and common models include the autoregressive integrated moving average (ARIMA) model [5] and the generalized autoregressive conditional heteroskedasticity (GARCH) model [6,7]. The method of machine learning can be divided into deep learning and classical machine learning methods such as support vector machines (SVM) [8,9]. In recent years, deep neural networks have been widely used in several fields due to their good feature extraction ability [10,11,12]. Meanwhile, it has also become a common method in network traffic prediction. Miguel [13] used artificial neural network swarms to predict long-term internet flow. They presented four network flow prediction models that are based on the ensemble of time-lagged feedforward networks (TLFNS), demonstrating the superiority of the proposed models by comparing them to the classical Holt–Winters approach. Nie [14] fused deep belief networks and Gaussian models, extracted the low-pass components of network traffic that can describe its own long-distance dependence using discrete wavelet transform and then learned deep belief networks from the low-pass components to build prediction models. Fang [15] used graph convolutional neural networks and long short-term memory (LSTM) to capture the temporal and spatial aspects of the cellular network of a single cell and build a prediction model, respectively. Zhang [16] proposed a spatio-temporal graph convolutional gated recurrent unit (GC-GRU) model to capture the spatial features of network traffic using graph convolutional neural network (GCN) and further process the spatio-temporal characteristics features using gated units (GRU) to improve the prediction performance of network traffic.

Deep models usually have complex structures and large parameters, so these models require repeated iterations to train the network, spending long training time and computational resources. Chen [17] proposed a new shallow neural network based on random vector function-link neural networks (RVFLNN) [18,19,20], named as broad learning system (BLS). It contains only one hidden layer, consisting of feature mapping nodes and enhancement nodes, which reduces the complexity of the neural network and has some feature capturing ability. Meanwhile, Chen proposed an incremental learning algorithm to calculate the output weights of the newly added hidden layer nodes in the BLS, so it can complete the training of the model in a shorter time while obtaining better accuracy [17,21,22]. Since its proposal, BLS has received a lot of attention and gained rapid development [23,24]. The experiment of Chen using BLS to predict short-term wind power has demonstrated it has good performance in time-series prediction [25]. However, as a kind of neural network, the hyperparameters of the BLS have a large impact on the network accuracy, and researchers usually need to train the model repeatedly to adjust the network hyperparameters to improve the model precision. This manual tuning method not only consumes a lot of time and energy but also wastes resources such as electricity for repeated training. Therefore, automatic hyperparameter optimization methods represented by population intelligence optimization algorithms such as ant colony algorithms [26] and particle swarm algorithms [27] have been developed as a result. In recent years, there have been numerous studies on the optimization of hyperparameters using population intelligence optimization algorithms. Zhou [28] improved the gray wolf algorithm and used this optimization algorithm to optimize hyperparameters such as kernel parameters in support vector machines. Xu [29] used the whale optimization algorithm to optimize the learning rate, training time, and the number of nodes in two hidden layers of the BiLSTM_Attention model to maximize the performance of the model. The ant colony algorithm and particle swarm algorithm have problems such as being easy to fall into local optimum and unsuitable for convergence. Sparrow search algorithm (SSA) [30] is a new type of swarm intelligence optimization algorithm with the advantages of good stability, strong global search ability, and fast convergence, so it has attracted extensive attention and research from scholars at home and abroad [31,32]. Tian [33] used SSA to optimize the hyperparameters of LSTM networks. Gai [34] used SSA to compute the best learning rate and batch size of deep confidence networks. Song [35] used SSA to optimize the penalty parameters and kernel function parameters of least squares support vector machines to improve the prediction accuracy and generalization ability of LSSVM.

In order to establish a fast and accurate network traffic prediction model, BLS is applied to network traffic prediction in this paper. At the same time, in order to quickly select the optimal hyperparameters to reduce their influence on the accuracy of the BLS, this paper combines the SSA with BLS, uses SSA to filter out the optimal combination of hyperparameters, and then uses the optimal hyperparameters to train BLS to build the network traffic prediction model.

The remaining part of this paper is organized as follows. Section 2 introduces the relevant methods used, including the BLS and the SSA. Section 3 introduces the proposed broad learning system based on sparrow search algorithm (SSA-BLS). Section 4 presents our experiments: SSA-BLS model is trained using two public datasets and real traffic data collected from the switch interface of an enterprise cloud platform, and its performance is compared with other models to verify the performance of the model. Section 5 summarizes our work, presents the limitations of the current approach, and briefly describes future work.

## 2. Related Work

### 2.1. Broad Learning System (BLS)

**Figure 1:**
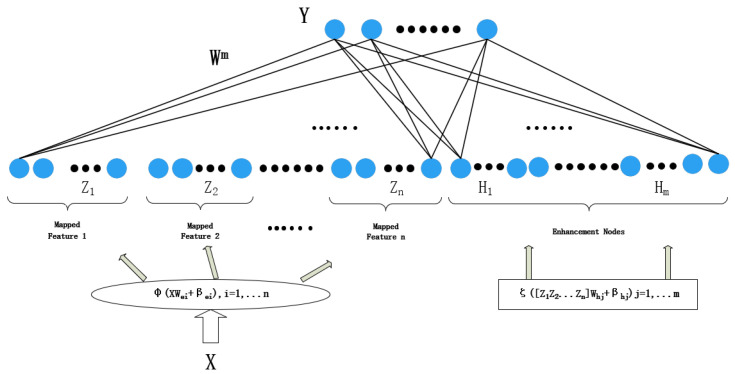
Broad learning system structure.

Broad learning system (BLS) is a new kind of shallow neural network based on the random vector functional-link neural network that is primarily used to tackle the problems of large computation and long training time for deep learning [17]. As shown in Figure 1, the hidden layer of the BLS is a single-layer structure, consisting of a feature mapping layer and an enhancement node layer.

The input training data $X\in R^{M\times N}$ of BLS contains M samples, each with N dimensions, and the corresponding label is $Y\in R^{M\times C}$. The feature mapping layer maps the input data into n sets of feature mappings with $K_{i}$ nodes by the feature mapping functions $\varphi_{i\;}\left(i=1,\ldots ,n\right)$:

(1) $$Z_{i}=\varphi_{i}(XW_{ei}+\beta_{ei}),i=1,2,\ldots ,n$$

where $Z_{i}$ means i-th group feature mapping; $W_{ei}$ and $\beta_{ei}$ is the randomly generated optimal feature mapping weight matrix and bias matrix, determined by the sparse self-encoder. In practical applications, $\varphi_{i}$ is often a nonlinear mapping function, such as Relu, Tanh. The groups of feature nodes are spliced to obtain the feature node matrix $Z_{in}=\left[Z_{1}Z_{2}\ldots Z_{n}\right]$. After that, the enhancement nodes are generated by the following equations:

(2) $$E_{j}=Z_{in}W_{hj}+\beta_{hj}$$

(3) $$H_{j}=\zeta_{j}\left(s\cdot E_{j}/\max E_{j}\right),j=1,2,\ldots ,m$$

The enhancement layer contains m groups and each consists of q nodes; $H_{j}$ denotes the j-th group of enhancement nodes; $W_{hj}$ and $\beta_{hj}$ are random weights and biases. $\zeta_{j}$ is a nonlinear activation function. Each group in the enhancement layer can choose a different $\zeta_{j}$ to fully extract the feature. In addition, s is the hyperparameter shrinkage coefficient.

Similarly, the m-group $H_{j}$ in the enhancement node layer is denoted as $H_{jm}=\left[H_{1}H_{2}\ldots H_{m}\right]$. Combine $Z_{in}$ and $H_{j}$ to obtain the hidden layer A:

(4) $$A=(Z_{in}|H_{jm})$$

Then, the label of training data can be represented as:

(5) $$Y=\left(Z_{in}|H_{jm}\right)W=\mathrm{AW}$$

where W is the weight of the output layer connected to the hidden layer, that is the network parameters to be learned, which can be calculated by the matrix pseudo-inverse:

(6) $$W=A^{†}Y$$

(7) $$A^{†}=\underset{\lambda \to 0}{\lim}{\left(\lambda I+A^{T}A\right)}^{-1}A^{T}$$

where $A^{†}$ is the pseudo-inverse matrix of matrix A; I is a unit matrix, and λ > 0 is a hyperparameter, regularization coefficient.

**Figure 2:**
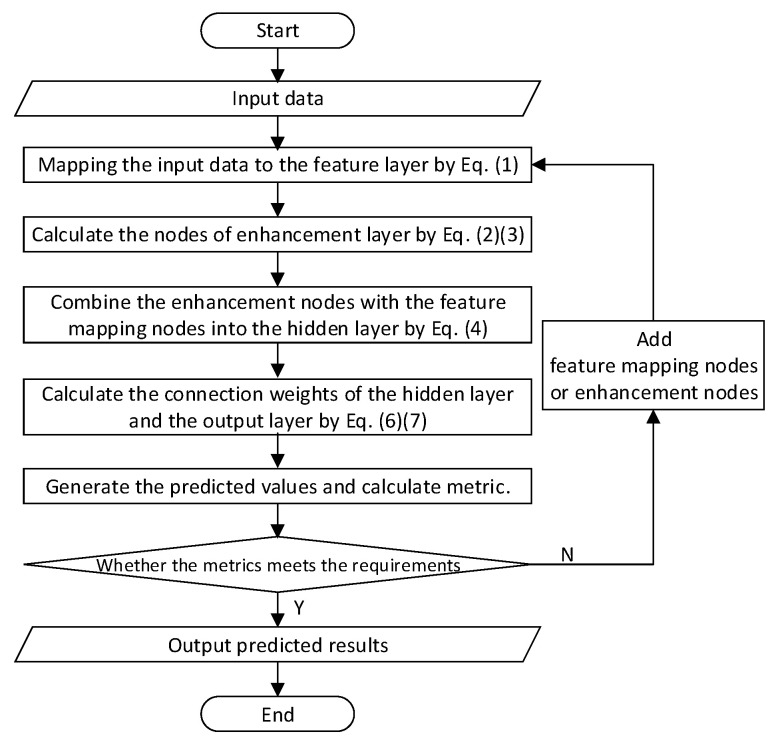
The algorithm flow chart of broad learning system.

BLS has two key characteristics compared with the deep neural networks. First, to better represent the input data and enhance computing efficiency, it employs sparse self-encoders to filter the random features of the input data into sparse and compact feature sets, then mines the key features using sparse feature learning models. Secondly, it addresses the problem that if the network model cannot reach the required accuracy in the deep learning system, it needs to add network layers or retrain the network after changing the structure. BLS employs the incremental learning algorithm to dynamically adjust the model by adding hidden layer nodes, which can obtain great accuracy in a short time. Figure 2 shows the algorithm flow chart of BLS.

### 2.2. Sparrow Search Algorithm (SSA)

Sparrow search algorithm is a new method of swarm intelligence optimization that is relied on sparrows’ predatory and anti-predatory behavior [30]. It divides sparrows into explorers and followers and designs the following rules according to the sparrow’s movement patterns.

(1) Similar to the slap swarm algorithm [36], sparrows in the population are divided into explorers and followers according to their fitness. The fitness is the objective function of optimization, which reflects the quality of the sparrow’s position.
(2) Sparrows with good fitness are explorers, and others act as followers. The explorer is responsible for investigating food-rich locations and guiding the followers to foraging locations and directions. The followers were able to search for the explorer with the best feeding position and then foraged around it.
(3) The fitness of a sparrow is dynamic, so the identity of explorers and followers can change with each other, but the proportion of explorers remains the same.
(4) The bad fitness of the followers, the worse their foraging position is indicated. These followers may randomly fly to other places to forage.
(5) A certain percentage of individuals in the sparrow population was selected as scouter, responsible for monitoring the safety of their surroundings. When a predator is detected, the scouter will sound an alarm, and when the alarm value is bigger than the safety value, the explorer will lead the followers to a safer area to forage.
(6) When danger is recognized, sparrows located at the edge of the group will quickly move to a safe area to get a better position, while sparrows located in the center will move randomly.

**Figure 3:**
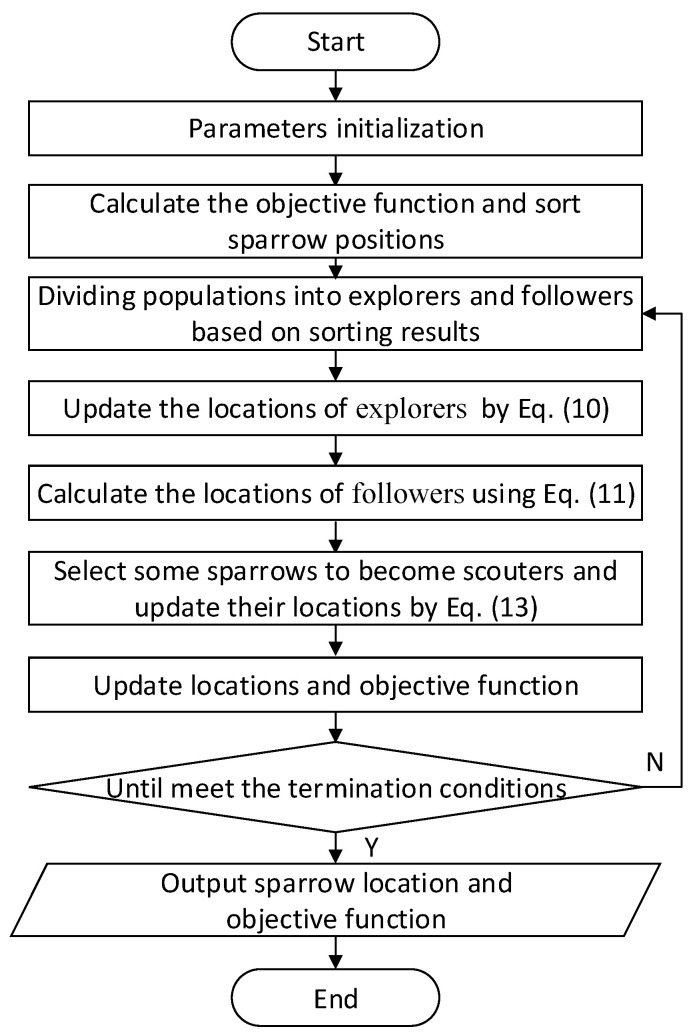
The algorithm flow chart of sparrow search algorithm.

The steps of SSA are as follows, and its algorithm flow chart is shown in Figure 3.

Step 1: Parameter initialization, which mainly includes setting the number of sparrow population, the proportion of explorers, the location of sparrows, and the number of iterations. The population containing n sparrows can be expressed as:

(8) $$X=\left[\begin{array}{ccc} x_{1}^{1} & x_{1}^{2} & \begin{array}{cc} \ldots & x_{1}^{d} \end{array}\\ x_{2}^{1} & x_{2}^{2} & \begin{array}{cc} \ldots & x_{2}^{d} \end{array}\\ \begin{array}{c} \vdots \\ x_{n}^{1} \end{array} & \begin{array}{c} \vdots \\ x_{n}^{2} \end{array} & \begin{array}{cc} \begin{array}{c} \vdots \\ \ldots \end{array} & \begin{array}{c} \vdots \\ x_{n}^{d} \end{array} \end{array} \end{array}\right]$$

where $x_{n}^{d}$ denotes the position of the n-th sparrow in dimension d; n is the population size, and d is the dimension of the variable to be optimized.

Step 2: Calculate the objective function and sort the sparrow positions. The objective function of the i-th sparrow can be indicated as:

(9) $$F_{i}=f\left(\left[\begin{array}{ccc} x_{i}^{1} & x_{i}^{2} & \begin{array}{cc} \ldots & x_{i}^{d} \end{array} \end{array}\right]\right)$$

where f denotes the objective function.

Step 3: Determine whether the current position of the population is safe and update the explorers’ position:

(10) $$X_{ij}^{t+1}=\{\begin{array}{l} X_{ij}^{t}\cdot \exp\left(\frac{-i}{\alpha \cdot b}\right)\;,R_{2}<ST\\ X_{ij}^{t}+QL\;\;\;\;\;\;\;\;\;\;\;,R_{2}\geq ST \end{array}$$

where $X_{ij}^{t}$ denotes the value of the i-th sparrow in the t-th iteration in the j-th dimension; b is a constant whose value is the maximum number of iterations; $R_{2}$ denotes the warning value in the range of [0, 1], which is a uniformly distributed random number; ST denotes the safety threshold and takes the value range of [0.5, 1.0]; *L* is a 1×d dimensional matrix; Q is a random number subjecting to normal distribution. When $R_{2}<ST$ means that the current location is safe and the sparrow flock goes to look for food. Conversely, the current location is threatened and the explorer needs to guide the sparrow flock to look for a new place to find food.

Step 4: Determine the state of the follower and update its position. The location is updated as follows:

(11) $$X_{ij}^{t+1}=\{\begin{array}{l} Q\cdot \exp\left(\frac{X_{w}^{t}-X_{ij}^{t}}{i^{2}}\right)\;\;\;\;\;\;\;\;\;\;\;\;\;\;\;\;\;\;,i>n/2\\ X_{p}^{t+1}+\left|X_{ij}^{t}-X_{p}^{t+1}\right|\cdot A^{+}\cdot L\;,i\leq n/2 \end{array}$$

(12) $$A^{+}=A^{T}{\left(AA^{T}\right)}^{-1}$$

where $X_{w}$ denotes the worst position in the sparrow population; $X_{p}$ is the position of the optimal explorer; A is a 1×d dimensional matrix with each dimensional value randomly generated from [−1, 1]. When i>n/2, it means that the follower is poorly positioned and does not get food, it needs to go to other places where it can get more food. Conversely, it continues to search for food near the explorer.

Step 5: Some sparrows find the danger and become scouters, updating the location of the scouters as follows:

(13) $$X_{ij}^{t+1}=\{\begin{array}{l} X_{b}^{t}+\beta \cdot \left|X_{ij}^{t}-X_{b}^{t}\right|\;\;\;\;\;\;,f_{i}>f_{g}\\ X_{ij}^{t}+K\cdot \left(\frac{\left|X_{ij}^{t}-X_{w}^{t}\right|}{\left(f_{i}-f_{w}\right)+\varepsilon }\right)\;,f_{i}=f_{g} \end{array}$$

where $X_{b}$ denotes the best position in the population; $f_{i}$ is the objective function of i-th sparrow, $f_{g}$ is the best value of the objective function, and $f_{w}$ is the worst value of the objective function; β is a standard normally distributed random number; K is a uniform random number with values in the range of [−1, 1]; ε is a smaller value to prevent the denominator from being zero.

Step 6: Update the objective function.

Step 7: Determine whether it satisfies the iteration stop condition and, if not, repeat steps 3 to 6.

## 3. Broad Learning System Based on the Sparrow Search Algorithm (SSA-BLS)

To minimize the impact of network hyperparameters and improve the accuracy of network flow forecasting, this paper employs the sparrow search algorithm to optimize hyperparameters of the broad learning system, shrinkage coefficient (r), and regularization coefficient (λ), to obtain the optimal hyperparameters and use them to build the training model. We named this method Sparrow Search Algorithm-Broad Learning System (SSA-BLS), and the algorithm is broken down into five steps.

Step 1: Parameter initialization. Determine the parameters of SSA, for example, explorer proportion and population size. Determine the range of shrinkage coefficient (r) and regularization coefficient (λ), respectively, and generate p (p is the population size) groups of initial hyperparameters as the initial position of the sparrow. The sparrow population is expressed as:

(14) $$X=\left[\begin{array}{cc} r_{1} & \lambda_{1}\\ r_{2} & \lambda_{2}\\ \begin{array}{c} \vdots \\ r_{p} \end{array} & \begin{array}{c} \vdots \\ \lambda_{p} \end{array} \end{array}\right]\;$$

where r and λ are randomly generated. They are the hyperparameters to be optimized.

Step 2: Choosing the root mean square error (RMSE) of the BLS’s prediction as to the objective function. Using the p sets of initial hyperparameters generated in the first step trains BLS to obtain the initial objective function. The objective function of the i-th sparrow is calculated as follows:

(15) $$f_{i}=\sqrt{\frac{\sum_{j=1}^{n}{\left({\hat{y}}_{j}^{i}-y_{j}\right)}^{2}}{n}},i=1,2,\ldots ,p$$

where ${\hat{y}}_{j}^{i}$ is the predicted value of the j-th sample of the BLS trained with the i-th set of hyperparameters; $y_{j}$ is the true value of the j-th sample; n is the number of training samples. The smaller the $f_{i}$, the better.

Step 3: The objective function is input into SSA, and execute the algorithm. According to the algorithm to update the sparrow population and objective function to achieve optimization of the hyperparameters of BLS.

Step 4: If the predefined number of iterations is reached, the optimization is completed and output the minimum value of the objective function:

(16) $$f_{m}=\min\left(\left[f_{1},f_{2},\ldots ,f_{p}\right]\right)$$

where m is the subscript of the minimum objective function. Then the hyperparameters obtained by SSA are:

(17) $$r,\lambda =x_{m}=\left[\begin{array}{cc} r_{m} & \lambda_{m} \end{array}\right]$$

Step 5: Put the hyperparameters r and λ obtained in the previous step into the BLS, train and build the network flow prediction model.

**Figure 4:**
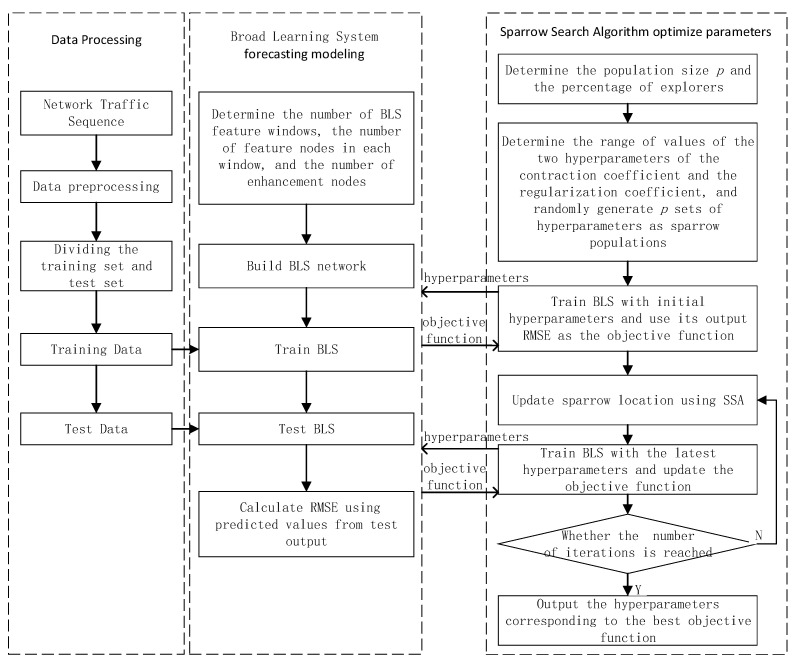
The algorithm flow chart of SSA-BLS.

The SSA-BLS flow chart is given in Figure 4.

## 4. Experimentation

All the experimental programs are developed based on python 3.8, the main packages used include numpy1.21 and pandas1.3, and the deep learning related models are implemented using pytorch3.8. The experimental environment is Windows 10OS, Intel (R) Core (TM) i5-1135G7 2.40GHz CPU, and 16.0GB RAM.

### 4.1. Datasets

The experimental dataset uses the core network traffic dataset of European cities and the academic backbone network traffic dataset of the UK.

The core network traffic dataset of European cities: the traffic of 11 European cities in the private ISPs. The traffic in bits on a transatlantic link from 7 June 2005, at 06:57 to 31 July 2005, at 11:17 are collected with a sampling interval of five minutes.

UK academic backbone traffic dataset: the dataset collects gathering flow from UK academic network backbone from 09:30 on 19 November 2004, to 11:11 on 27 January 2005, with a sampling interval of five minutes.

We use the data from 1 July to 25 July 2005, in the core network traffic dataset of European cities as the training set and from 26 July to 28 July as the test set; the data from 1 January to 24 January 2005, in the UK academic network backbone traffic dataset as the training set and the test set is from 25 January to 27 January.

### 4.2. Parameters and Evaluation Indicators

The SSA-BLS parameters are chosen as follows. Setting population size as 50, the explorer proportion is 20%, and the maximum number of iterations is 5; the number of windows in the mapping layer is 10, the number of nodes within each window in the feature mapping layer is 10, the enhancement nodes’ number is 50, and the values of shrinkage coefficient (r) and regularization coefficient (λ) are taken in the ranges of [0.09, 0.999999] and [2^−30^, 2^−35^], respectively.

Mean squared error (MSE), root mean square error (RMSE), mean absolute error (MAE), mean absolute percentage error (MAPE), and moving average (MA) are used as evaluation indicators. These indicators are calculated as follows:

(18) $$\mathrm{MSE}=\frac{1}{n}\overset{n}{\underset{i=1}{\sum }}{\left({\hat{y}}_{i}-y_{i}\right)}^{2}$$

(19) $$\mathrm{RMSE}=\sqrt{\frac{1}{n}\overset{n}{\underset{i=1}{\sum }}{\left({\hat{y}}_{i}-y_{i}\right)}^{2}}$$

(20) $$\mathrm{MAE}=\frac{1}{n}\overset{n}{\underset{i=1}{\sum }}\left|{\hat{y}}_{i}-y_{i}\right|$$

(21) $$\mathrm{MAPE}=\frac{1}{n}\overset{n}{\underset{i=1}{\sum }}\left|\frac{{\hat{y}}_{i}-y_{i}}{y_{i}}\right|$$

(22) $$\mathrm{MA}=1-\mathrm{MAPE}=\left(1-\frac{1}{n}\overset{n}{\underset{i=1}{\sum }}\left|\frac{{\hat{y}}_{i}-y_{i}}{y_{i}}\right|\right)*100\%$$

where samples number is n, $y_{i}$ is the true value and ${\hat{y}}_{i}$ is the output. The smaller the MSE, RMSE, MAE, and MAPE, the better, while MA is closer to 100% indicating better model prediction performance.

### 4.3. Results and Discussion

The flow values of [T−11,T] were input into the SSA-BLS to predict the flow at the moment of T+1 in the experiment. We compare SSA-BLS with the similarly structured BLS, Extreme Learning Machine (ELM) [37], Stochastic Configuration Networks (SCN) [38], RVFLNN, dRVFL (deep RVFL) (the variant of RVFL) [39], and the LSTM [40] that is commonly used in network traffic prediction and used to evaluate the quality of the SSA-BLS. Each model is run 100 times independently, and the prediction metrics of different models are evaluated individually each time, taking the average metrics as the final result of each model. The following are the parameters of each model: the values of r and λ for BLS are automatically obtained from {0.1, 0.5, 0.9, 0.99, 0.9999, 0.99999} and {2^−30^, 2^−20^, 2^−10^, 0.5, 1, 5, 10}, respectively, and the remaining parameters are the same as those of the SSA-BLS; the SCN can have a maximum of 250 hidden layer nodes, the training tolerance is 0.001, and candidate nodes maximum allowed 100; the regularization factor of RVFL is $1\times 10^{-3}$, and hidden layer has 100 nodes; dRVFL parameters are the same as RVFL; the hidden layer of ELM contains 200 nodes, and the maxing coefficient for distance and dot product input activations is 1.0; the LSTM contains 3 hidden layers, each with 12 blocks, and is trained with a learning rate of $1\times 10^{-2}$, batch size of 64 and epoch is 15. Table 1 and Table 2 show the prediction performance of two datasets on the different models.

**Figure 5:**
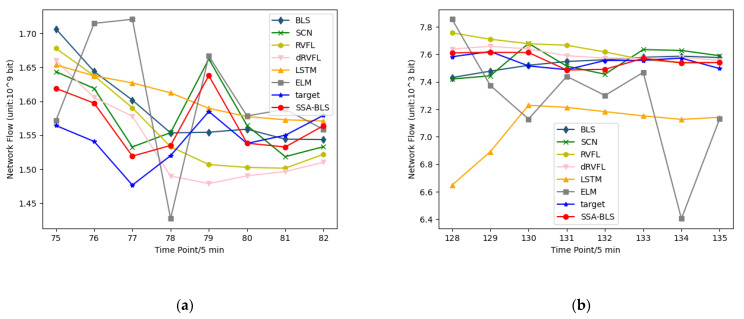
(**a**) Prediction results for a core network traffic dataset in European cities; (**b**) UK academic backbone network traffic dataset forecast results.

On the test set of the public dataset, Figure 5 shows the predicted versus true values of the SSA-BLS model versus the other models. Moreover, to better validate the prediction accuracy of the SSA-BLS model, the model is applied to a private traffic dataset. The private traffic dataset is derived from the real incoming traffic data of switch interfaces of an enterprise from 5 October to 18 October 2021. We employ the data from 5 October to 16 October 2021, in the private dataset as training data, using the data from 17 October to 18 October as test data.

Since the sampling interval of the enterprise switch interface traffic data is unequal, the resampling is first performed: the average value of the interface traffic within 5 min is calculated, and if there is no traffic data within 5 min, the previous value is used to fill in. Meanwhile, there are great abnormal traffic values in the original data, and to lessen the influence of abnormal values, the data are smoothed using spectral smoothing (spectral smoother). Table 3 shows the experimental results.

**Figure 6:**
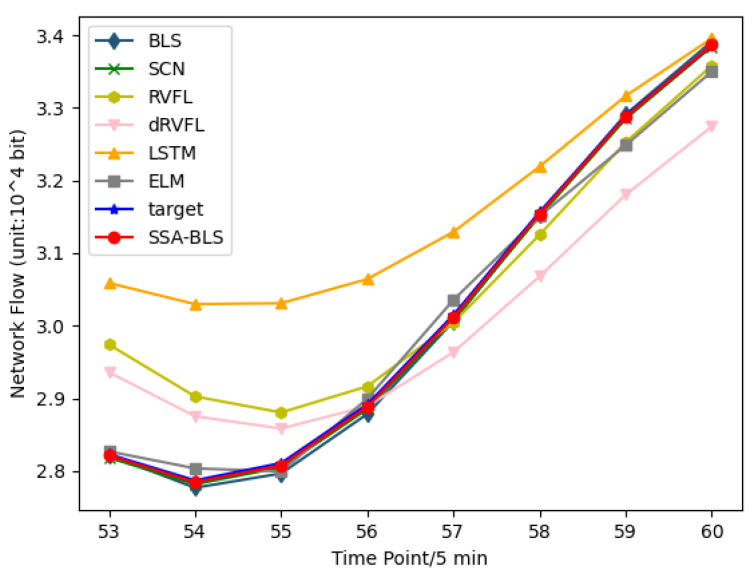
Enterprise cloud platform switch interface traffic dataset prediction results.

Figure 6 shows the predicted versus true values of the SSA-BLS compared to others on the test data in the private dataset. It is clear from Table 1, Table 2 and Table 3 that the hyperparameters have a strong influence on BLS. If the hyperparameters are bad, the prediction performance of BLS will be degraded. The results show that the SSA-BLS model has better prediction accuracy than the other models on both the UK academic backbone network traffic dataset and the enterprise cloud platform switch interface traffic dataset, and its prediction performance on the European urban core network traffic dataset is only slightly below SCN. It can be seen that the SSA-BLS model, which is obtained after optimizing BLS using SSA, provides optimal hyperparameters for BLS through SSA, so that the SSA-BLS model can choose to capture the time characteristics of traffic better, and its network traffic prediction capability gains a large improvement compared with the original BLS model.

**Figure 7:**
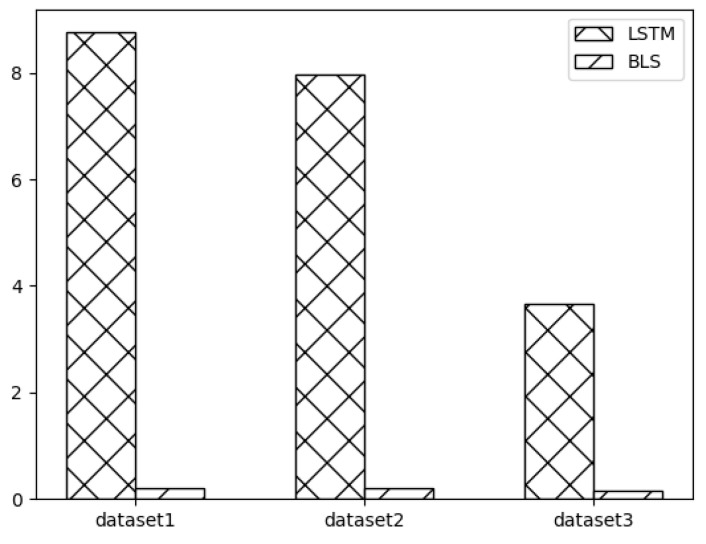
BLS and LSTM runtime.

Meanwhile, this paper uses BLS for network traffic prediction based on the advantage of less training time due to its “expanding landscape” network structure. The running time of BLS in SSA-BLS is the main factor affecting the time of SSA-BLS. In order to verify the advantage of SSA-BLS model in time consumption, we compare the running time of BLS and the running time of LSTM for one epoch on three datasets, and the experimental results are shown in Figure 7. In Figure 7, dataset 1, dataset 2, and dataset 3 are the UK academic backbone network traffic dataset, European urban core network traffic dataset, and enterprise cloud platform switch interface traffic dataset, respectively. The experimental results show that BLS can complete the training in a shorter time, and the larger the data volume, the greater the advantage of BLS.

## 5. Conclusions, Limitations, and Future Research

Predicting future traffic on the cloud platform interface can be used to assist the cloud platform in provisioning resources and planning the network, and it is an effective way to help achieve dynamic resource allocation and load balancing with the volume of requests. In this paper, we propose a model named SSA-BLS to predict the network interface traffic. The model uses SSA to optimize two hyperparameters in BLS to obtain the optimal combination of hyperparameters quickly and enhance the performance of BLS. At the same time, the model uses BLS to capture the traffic timing features and reduce the training time of the prediction model. Finally, we apply SSA-BLS to the short-term network traffic prediction, selecting two public datasets of network traffic and a real dataset of network switch interface traffic of an enterprise cloud platform for experiments. Finally, we compare the SSA-BLS with other models, and the experiments show that the SSA-BLS can select better hyperparameters to make the network traffic prediction accuracy above 97%.

Currently, most network traffic prediction models have a strict sampling interval for training data, requiring the data to be equally spaced. Sometimes, frequent sampling is required to obtain more fine-grained data. However, frequent sampling for a long time will increase resource consumption, and it is difficult to present the data with equal spacing due to the inevitable packet loss during the network transmission. Therefore, future research will be conducted for the prediction modeling of non-equally spaced sampled data to reduce the requirement of data spacing and improve the generalizability of the model.

## Figures and Tables

**Table 1:** Experimental results of a core network traffic dataset in European cities.

	MSE	RMSE	MAE	MAPE	MA
SSA-BLS	0.0159047	0.1261069	0.0937315	0.0294284	97.057155%
BLS	0.0781227	0.2551322	0.1878021	0.0571019	94.289801%
SCN	0.0154907	0.1244372	0.0934485	0.0295662	97.043378%
RVFL	0.0254023	0.1593589	0.1208186	0.0388347	96.116525%
dRVFL	0.0227553	0.1507691	0.1135728	0.0367191	96.328085%
ELM	0.1394488	0.3686439	0.2710739	0.0780252	92.197470%
LSTM	0.0781441	0.2502372	0.1884968	0.0517535	94.824642%

**Table 2:** Experimental results of UK academic backbone network traffic dataset.

	MSE	RMSE	MAE	MAPE	MA
SSA-BLS	0.0071345	0.0844570	0.0618339	0.0136258	98.637411%
BLS	0.0913392	0.2639297	0.1873060	0.0440354	95.596458%
SCN	0.0097822	0.0982373	0.0668127	0.0143890	98.561099%
RVFL	0.0289572	0.1701013	0.1179645	0.0264767	97.352324%
dRVFL	0.0234114	0.1527494	0.1058797	0.0232484	97.675151%
ELM	0.1051171	0.3157292	0.2166875	0.0426738	95.732612%
LSTM	0.3192739	0.3290859	0.2518907	0.0595686	94.043138%

**Table 3:** Enterprise cloud platform switch interface traffic data set experimental results.

	MSE	RMSE	MAE	MAPE	MA
SSA-BLS	0.0000734	0.0082991	0.0063628	0.0021407	99.785924%
BLS	0.0103714	0.0811761	0.0563080	0.0176119	98.238804%
SCN	0.0001742	0.0130396	0.0067544	0.0021857	99.781427%
RVFL	0.0361230	0.1899801	0.1288009	0.0400804	95.991952%
dRVFL	0.0327578	0.1807739	0.1277397	0.0403579	95.964208%
ELM	0.0579519	0.2382928	0.1327614	0.0400340	95.996599%
LSTM	0.0283041	0.1057008	0.0759722	0.0238097	97.619024%
